# Severe *Chlamydia psittaci* pneumonia complicated by deep vein thrombosis: a case report and literature review

**DOI:** 10.3389/fmed.2026.1865706

**Published:** 2026-07-28

**Authors:** Mengdie Chen, Qing Miao, Lina Fu, Shuhua Rong, Zhaoyun Peng, Kejing Zheng, Yuxia Liu, Min Wang

**Affiliations:** 1 Shandong University of Traditional Chinese Medicine, Jinan, China; 2 The Changqing District People’s Hospital, Jinan, China; 3 The Second Affiliated Hospital of Shandong University of Traditional Chinese Medicine, Jinan, China

**Keywords:** *Chlamydia psittaci*, *Chlamydia psittaci* pneumonia, deep vein thrombosis, literature review, severe pneumonia

## Abstract

Psittacosis is a zoonotic disease caused by *Chlamydia psittaci*, primarily transmitted through the respiratory tract. In humans, the most frequently associated condition is atypical pneumonia, while *Chlamydia psittaci* remains a relatively uncommon pathogen in cases of community-acquired pneumonia. Historically, reports of pneumonia due to *Chlamydia psittaci* have been limited, largely due to challenges in diagnosis. However, recent advancements in technologies such as next-generation sequencing (NGS) have led to an annual increase in reported cases of *Chlamydia psittaci* infection. Despite this, many hospitals still possess limited knowledge regarding *Chlamydia psittaci*, particularly in the context of diagnosing and treating critically ill patients. This report presents a case of severe *Chlamydia psittaci* pneumonia complicated by deep vein thrombosis in a 50-year-old male patient. The diagnosis was confirmed through polymerase chain reaction (PCR), and the patient was successfully treated with omadacycline. By analyzing 55 cases, this paper aims to summarize the etiology, clinical manifestations, diagnostic approaches, and treatment strategies for severe *Chlamydia psittaci* pneumonia, thereby enhancing clinicians’ understanding of this disease.

## 1 Introduction

Amid the growing trend of pet ownership and the increasing diversity of human-animal interactions, *Chlamydia psittaci* pneumonia, a well-known zoonotic disease, has experienced a resurgence of sporadic cases globally in recent years. Some regions have reported cluster outbreaks and severe cases, prompting renewed interest from both clinical and public health sectors. The disease is caused by infection with *Chlamydia psittaci*, a pathogen that can infect over 190 species of birds and poultry, including parrots, pigeons, chickens, and ducks. Humans are primarily infected through inhalation of aerosols containing pathogens or direct/indirect contact with excreta and feathers of diseased poultry (1). The clinical manifestations of *Chlamydia psittaci* pneumonia are often non-specific, typically beginning with high fever and cough, which can easily be mistaken for common respiratory illnesses such as influenza, mycoplasma pneumonia, or Coronavirus Disease 2019 (COVID-19). The incubation period varies from 3 to 45 days, and some patients may experience severe complications, including liver and kidney dysfunction and respiratory failure, resulting in a significant rate of missed and misdiagnosis (2). Research indicates that middle-aged and elderly individuals, immunocompromised persons, and those working in poultry breeding or contact are at heightened risk, with the mortality rate for severe cases lacking timely and standardized treatment potentially reaching 10–20% (3). In light of the current epidemiological characteristics and clinical challenges associated with this disease, this study first presents the complete clinical data of a confirmed case of *Chlamydia psittaci* pneumonia complicated by deep vein thrombosis. Subsequently, it conducts a search of the PubMed database using the keywords “psittacosis” and “*Chlamydia psittaci* pneumonia” to identify English case reports published between 2012 and 2025 for literature analysis. The objective is to systematically delineate the clinical features, imaging manifestations, and critical diagnostic and treatment considerations, thereby providing a reference for clinicians to enhance early identification, accurate diagnosis, and effective management of this condition.

## 2 Case presentation

A 50-year-old male patient was admitted to the hospital on November 11, 2025, presenting with a cough and fever that had persisted for 10 days. He had been employed at a local cold storage facility, where he worked slaughtering ducks for two weeks. Ten days prior to admission, he experienced a paroxysmal dry cough, accompanied by fever (peak temperature 39.7 °C), night sweats, occasional chest tightness, shortness of breath, and pain in the left lower limb. He received intramuscular antipyretic medications at a local clinic (details not specified), which proved ineffective. On November 8, the patient was admitted to a local hospital where comprehensive laboratory tests and examinations were performed. After receiving symptomatic treatment, the patient was discharged on their own initiative. Persistent symptoms included paroxysmal cough, fever, chest tightness, dyspnea, and pain in the left lower limb. For further diagnosis and treatment, the patient visited the outpatient department of the Second Affiliated Hospital of Shandong University of Traditional Chinese Medicine and was admitted to our department with a diagnosis of “severe pneumonia.”

Upon admission, the patient’s vital signs were as follows: temperature 36.5 °C, heart rate of 96 bpm, respiratory rate 19 breaths / minute, and blood pressure 126/83 mmHg. He exhibited mild dyspnea appearance, cyanotic lips, slight pharyngeal congestion, coarse breath sounds bilaterally, and a small amount of moist rales in the right lower lung. There was no edema in either lower limb, although the patient reported pain and discomfort in the left lower limb. The patient denied a history of chronic conditions such as hypertension, coronary heart disease, diabetes mellitus, chronic kidney disease, or chronic bronchitis, and had no smoking history.

On November 8, lower extremity arterial and venous color Doppler ultrasound was performed at the local hospital: bilateral femoral vein valve insufficiency; bilateral intermuscular vein thrombosis. On November 9, cardiac color Doppler ultrasound revealed tricuspid regurgitation (mild).

**Figure 1:**
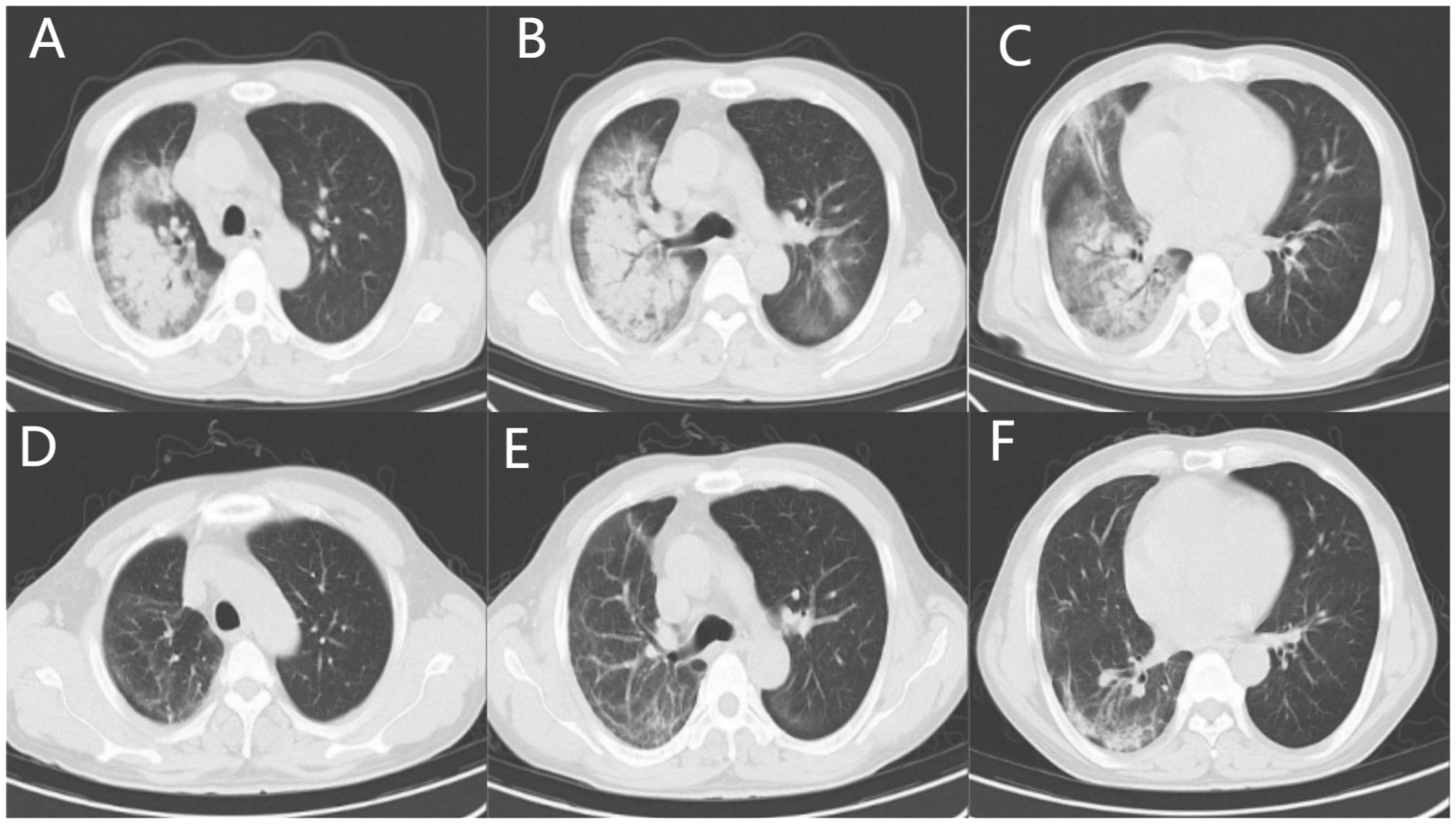
Chest computed tomography (CT) of the patient: The initial chest CT on admission showed dual pneumoniaous lesions with minimal right pleural effusion **(A–C)**. After 15 days of omadacycline treatment, the chest CT showed a reduction in pulmonary consolidation and pleural effusion **(D–F)**.

Laboratory tests conducted upon admission revealed an elevated white blood cell count, neutrophil count, D-dimer, and C-reactive protein (CRP) (Table 1). Procalcitonin (PCT) measured 0.956 ng/mL (normal range: 0–0.5 ng/mL), while interleukin-6 (IL-6) was recorded at 45.27 pg./mL (normal range: ≤7 pg./mL). Arterial blood gas analysis (the blood gas analysis specimen was collected when the patient was breathing ambient air at rest) showed pH 7.52, PO₂ 53 mmHg, PCO₂ 33 mmHg, and HCO₃ 26.9 mmol/L. The coagulation panel showed fibrinogen degradation products (FDP) at 66.63 μg/mL (normal value <5.0). Blood biochemistry results demonstrated a decreased albumin level. Electrolyte levels are presented in Table 1. Glucose and glycated hemoglobin tests showed glucose at 6.44 mmol/L (normal range: 3.9–6.1 mmol/L) and glycated hemoglobin at 6.1% (normal range: 4–6%). Cardiac infarction four-item test: B-type natriuretic peptide precursor (Pro-BNP) measurement: 899 pg./mL (normal range: 0–125), myohemoglobin: 72.41 ng/mL (normal range: 28–72). The cryptococcal capsular polysaccharide antigen test, along with influenza and COVID-19 nucleic acid tests and the *Mycoplasma pneumoniae* antibody test, all returned negative results. A chest plain CT scan (Figures 1A–C) revealed multiple scattered patchy areas of increased density with ill-defined borders are observed in both lungs, accompanied by partial consolidation and bronchial air spaces. A small amount of pleural effusion is noted in the right thoracic cavity. An abdominal ultrasound identified a hyperechoic nodule in the right hepatic lobe, suggestive of hemangioma.

**Table 1:** The patient’s laboratory test results.

Test parameter	On the day of admission (Nov 11)	After treatment (Nov 28)	Normal range
WBC count (×10^9^/L)	10.54	6.26	3.5–9.5
Neutrophil count (×10^9^/L)	9.70	4.52	1.8–6.3
Neutrophil %	92.0	72.3	40–75
Lymphocyte count (×10^9^/L)	0.36	1.14	1.1–3.2
Lymphocyte %	3.4	18.2	20–50
Hemoglobin (g/L)	134	126	130–175
Platelets (×10^9^/L)	253	125	125–350
CRP (mg/L)	169.2	0.9	0–8
D-dimer (mg/L)	14.73	1.31	0–0.55
BUN (mmol/L)	6.01	8.48	3.1–8
Creatinine (μmol/L)	59.4	49.2	57–97
ALT (U/L)	301	57	9–50
AST (U/L)	121	19	15–40
ALB (g/L)	31.3	31.8	40–55
K (mmol/L)	2.95	4.13	3.5–5.5
Na (mmol/L)	131.1	136.6	135–145
Cl (mmol/L)	92.6	99.5	99–110

WBC, white blood cell; CRP, C-reactive protein; BUN, blood urea nitrogen; ALT, alanine aminotransferase; AST, aspartate aminotransferase; ALB, albumin.

**Figure 2:**
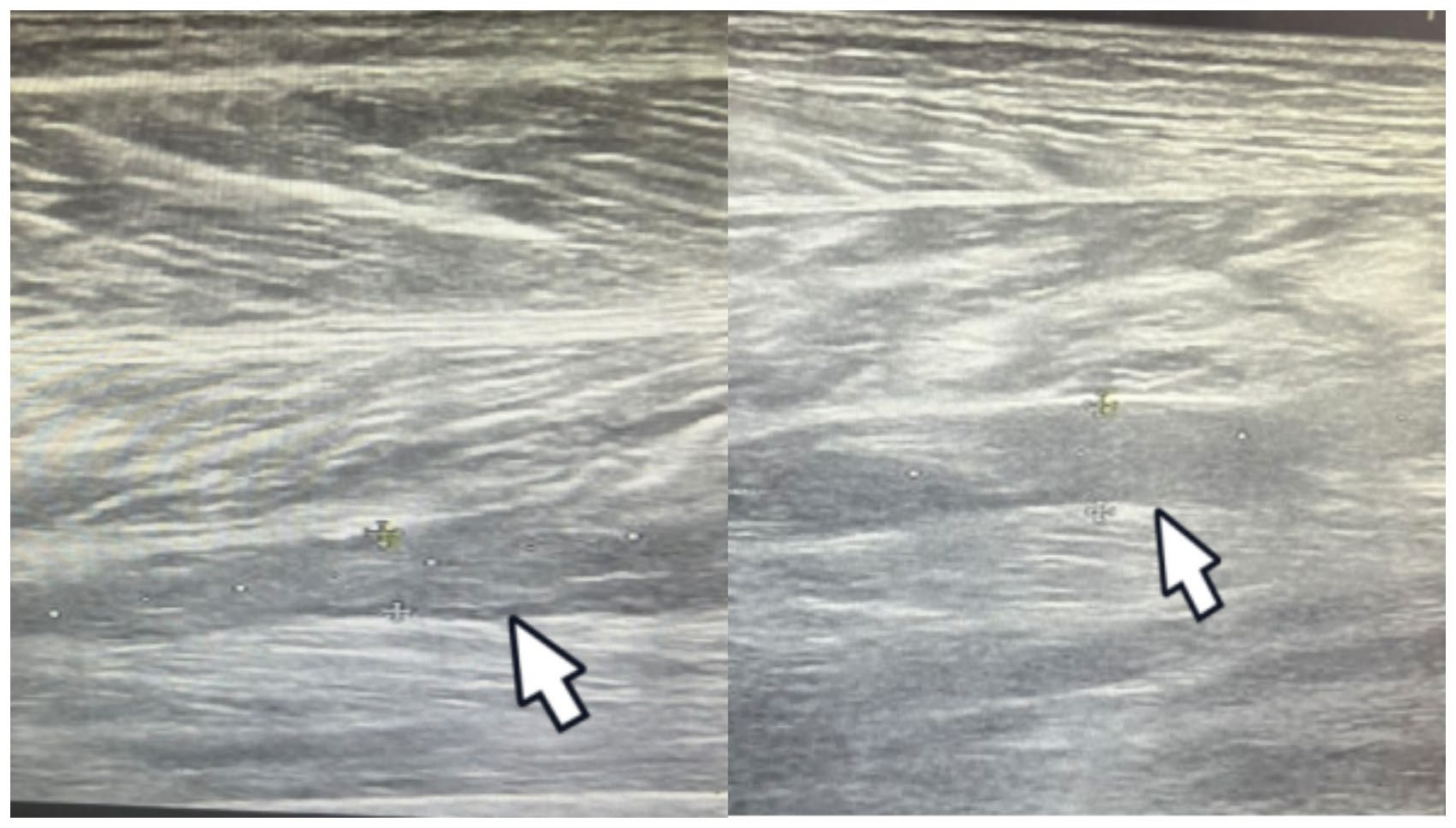
Venous ultrasound of the legs on admission showing thrombosis of the upper segment of the left tibial posterior vein (Left image) and bilateral intermuscular veins, with maximum dimensions of 5.2 × 0.6 cm on the left side and 6.2 × 0.9 cm on the right side.

Following hospital admission, the patient developed severe infection. Empirical anti-infective therapy was initiated with meropenem and omadacycline. The patient’s D-dimer level was markedly elevated to 14.73 mg/L (reference range: 0–0.55 mg/L), raising clinical suspicion of coagulation dysfunction. Although no obvious calf swelling was observed, bilateral lower extremity venous ultrasound was performed urgently. The results confirmed thrombosis in the upper segment of the left posterior tibial vein and bilateral intermuscular veins, with maximum thrombus dimensions of 5.2 × 0.6 cm (left) and 6.2 × 0.9 cm (right) (Figure 2).

Given the presence of lower limb thrombosis and coagulopathy, the patient was at high risk of pulmonary embolism and sudden death from thrombus detachment. Therefore, pulmonary artery computed tomography angiography (CTPA) was performed the next day, which ruled out pulmonary embolism. Subsequently, the patient was prescribed anticoagulant therapy with bemiparin sodium, placed on strict bed rest, and instructed to avoid lower limb massage. Nasal cannula oxygen therapy was administered at 3 L/min to maintain peripheral oxygen saturation above 95%,and it is recommended to perform lower extremity filter implantation as soon as possible.

**Figure 3:**
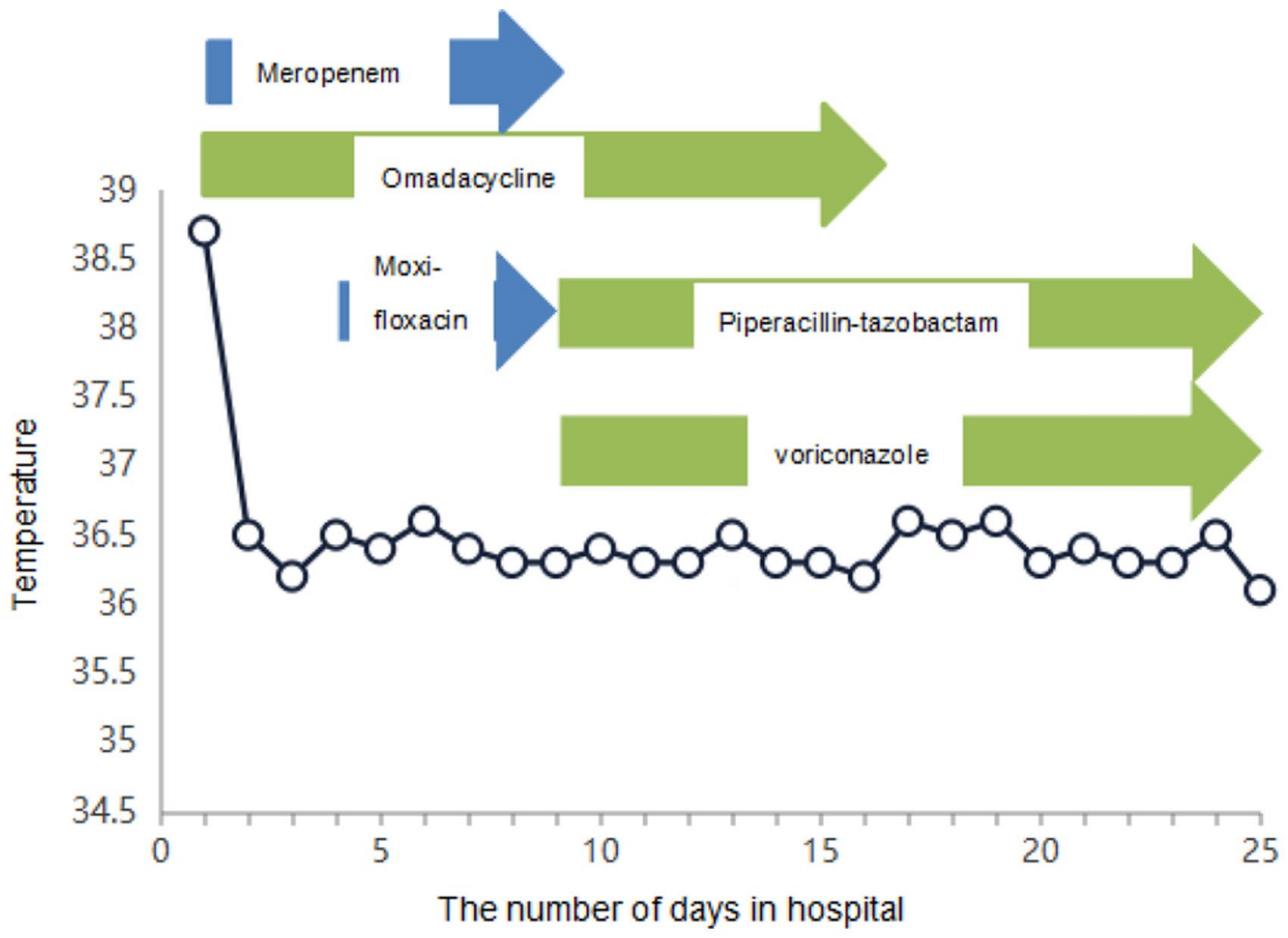
Body temperature and antimicrobial treatment during hospitalization at the Second Affiliated Hospital of Shandong University of Traditional Chinese Medicine.

Subsequently, the patient developed rapid-onset respiratory failure and liver injury. On the 2th day of admission, the patient became afebrile (Figure 3) but continued to experience paroxysmal cough, chest tightness, dyspnea, and left lower limb pain. Given the fulminant course of infection and the patient’s documented history of close poultry contact during cold chain processing, infection with atypical pathogens—including *Chlamydia psittaci* and avian influenza virus—could not be excluded. The initial antimicrobial regimen was continued, and comprehensive pathogenic sampling was performed. Early bronchoscopy with next-generation sequencing (NGS) was planned, and the hospital infection control department was contacted to coordinate specific testing for *C. psittaci* and avian influenza. On the same day, throat swab testing at the Jinan Center for Disease Control and Prevention returned positive for *C. psittaci*. Bronchoscopy was deferred due to the patient’s critical clinical status. Concurrent hypokalemia was corrected with oral potassium chloride supplementation, and magnesium isoglycyrrhizinate was continued for hepatoprotection in the setting of hepatic insufficiency.

On the 4th day of admission, the patient was transferred to the Department of Peripheral Vascular Medicine for IVC filter implantation to mitigate the risk of fatal PE from left lower limb DVT. On the 5th day of admission, the patient remained afebrile, with significant improvement in paroxysmal cough, chest tightness, and dyspnea. The patient exhibited poor glycemic control, which was managed with sliding-scale insulin based on point-of-care blood glucose measurements. Laboratory evaluation showed decreased C-reactive protein (CRP) levels, normal interleukin-6 (IL-6) and procalcitonin (PCT) levels, and improved liver function. The antimicrobial regimen was therefore adjusted: meropenem was de-escalated to 1 g every 12 h, while omadacycline was continued at the original dose.

On the 8th day of admission, sputum fungal culture grew sparse colonies of *Aspergillus flavus* and *Aspergillus fumigatus*, leading to a diagnosis of secondary invasive pulmonary aspergillosis. Voriconazole was initiated for targeted antifungal therapy. On the 12th day of admission, N-terminal pro-B-type natriuretic peptide (NT-proBNP) level normalized at 119 pg./mL. On the 15th day of admission, repeat chest computed tomography (CT) (Figures 1D–F) demonstrated multiple scattered ill-defined patchy opacities in both lungs, with focal consolidation and visible air bronchograms. Compared with prior imaging, the extent and density of pulmonary lesions were reduced, with localized linear and reticular infiltrates consistent with resolving inflammation. No pleural effusion was detected.

On the 20th day of admission, low-molecular-weight heparin was discontinued, and oral rivaroxaban was commenced to facilitate long-term anticoagulation. The patient’s clinical status stabilized on the 23th day of admission; intravenous corticosteroids were subsequently tapered and transitioned to oral prednisone acetate at a once-daily dose of 20 mg, with the patient maintained under continuous close clinical monitoring.

A repeat venous ultrasonography of the lower extremities performed on the 24th day of admission revealed complete recanalization of the thrombus within the proximal segment of the left posterior tibial vein, alongside partial resolution of bilateral intermuscular venous thrombosis. The patient was transferred to the Department of Peripheral Vascular Medicine the subsequent day for retrieval of the inferior vena cava (IVC) filter.

**Figure 4:**
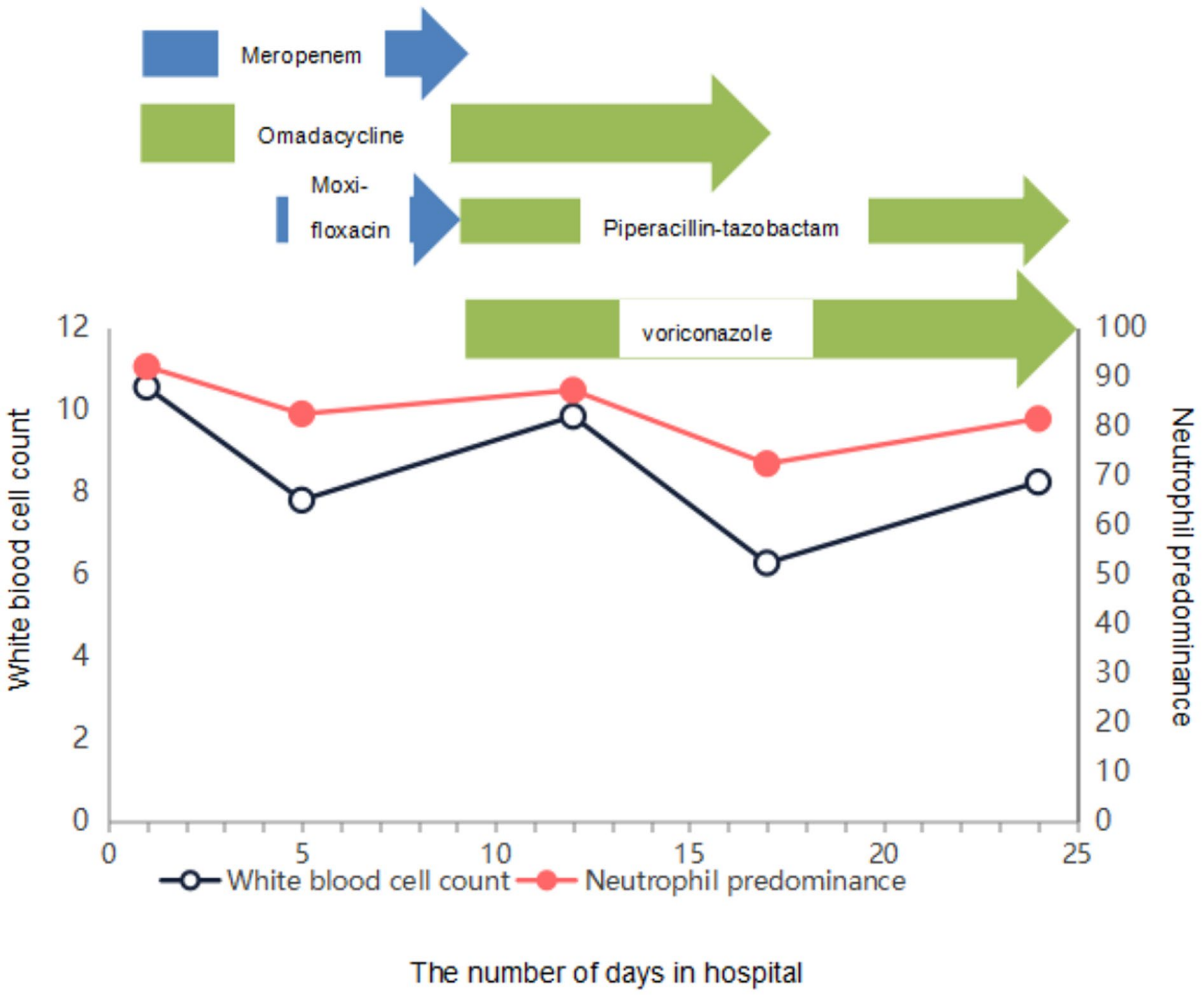
Change in white blood cell count and neutrophil predominance during hospitalization at the Second Affiliated Hospital of Shandong University of Traditional Chinese Medicine.

On the 26th day of admission, the patient exhibited markedly improved overall clinical status with substantial symptomatic relief and was discharged. Discharge medication and follow-up regimens were outlined as follows: oral rivaroxaban monotherapy to be continued for an additional month; prednisone acetate retained at 20 mg daily for 10 days, followed by a dose reduction of 5 mg every 10 days until full discontinuation; and voriconazole administered throughout the entire corticosteroid tapering course, combined with concomitant gastric mucosal protection and calcium supplementation. Scheduled surveillance protocols comprised serial hepatic and renal function assays, a chest computed tomography scan, and an outpatient respiratory clinic appointment at 2 months post-discharge. Blood glucose monitoring was recommended after corticosteroid withdrawal, with referral to endocrinology services for further assessment if clinically warranted.(Figure 4: Serial changes in white blood cell count and neutrophil percentage during hospitalization at the Second Affiliated Hospital of Shandong University of Traditional Chinese Medicine.)

## 3 Literature review

This study systematically searched the PubMed database for English case reports published between August 2012 and September 2025 using the search terms “*Chlamydia psittaci*” and “*Chlamydia psittaci* pneumonia.” Literature was strictly screened layer by layer according to predefined inclusion and exclusion criteria, with duplicate publications and repeated reports of the same patient excluded. A total of 54 eligible articles involving 54 cases were included; combined with an additional confirmed case of psittacosis caused by *Chlamydia psittaci* reported in this study, the final cohort comprised 55 cases, all of which are summarized in Supplementary Table 1.

Most cases reported a history of direct or indirect exposure, which included raising parrots, birds, or poultry, visiting bird shops, or passing through poultry markets. A small number of patients contracted the infection through contact with pets or aborted sheep, and instances of human-to-human transmission have also been documented. As summarized in Supplementary Table 1, the majority of cases (83.6%, 46/55) had a history of bird exposure, among which 3 deaths were reported. Symptoms reported in prior studies included fever (96.4%, 53/55), cough (87.3%, 48/55), myalgia (18.2%, 10/55), headache (21.8%, 12/55), and fatigue (25.5%, 14/55). Laboratory tests indicated that most patients exhibited a normal white blood cell count, with 27.3% (15/55) presenting with leukocytosis, 63.6% (35/55) with neutrophilia, 80% (44/55) with elevated CRP, and 61.8% (34/55) with elevated PCT. Chest X-ray or CT revealed abnormal findings in 54 patients (98.2%); another patient had incomplete imaging results due to insufficient medical records, while hilar lymphadenopathy (5.5%, 3/55) and pleural effusion (50.9%, 28/55) were observed infrequently. High-resolution CT identified solid nodules (5.5%,3/55), ground-glass opacities (21.8%, 12/55), air bronchograms (27.3%, 15/55). Additionally, there was one case of the reversed halo sign, one case of vacuolation sign and one case of migratory infiltration.

A total of forty-one patients (74.5%, 41/55) were administered quinolone therapy, which included moxifloxacin and levofloxacin. Six patients (10.9%, 6/55) received macrolide therapy, comprising azithromycin, erythromycin, and roxithromycin. Additionally, forty-four patients (80.0%, 44/55) underwent tetracycline therapy, either alone or in conjunction with quinolones or macrolides, utilizing doxycycline, minocycline, tigecycline, and omadacycline. Most cases resulted in a cure, with only three deaths reported (5.5%, 3/55).

## 4 Discussion and conclusion

*Chlamydia psittaci* is an obligate intracellular Gram-negative pathogen found in the tissues, blood, and feces of parrots and other avian species. *Chlamydia psittaci* primarily infects birds, but inhalation of contaminated aerosols produced by infected birds may occasionally cause psittacosis in humans. If the disease is not diagnosed or the pathogen identified promptly, and effective antibiotics are delayed, severe cases may result in high mortality rates, with a case fatality rate of 10–20% without timely and standardized treatment (3). Occasional outbreaks of psittacosis can pose a greater threat to society (4–6). Despite an increase in reported cases and heightened attention over the past decade, psittacosis remains a rare disease (4, 7). Due to low awareness of the disease and its diverse clinical manifestations, clinicians often struggle to identify psittacosis, particularly general practitioners. Therefore, it is necessary to conduct a discussion and review of psittacosis to enhance clinical awareness and management of this disease.

Most infected individuals are adults aged 30–60 years, with a majority having a history of contact with parrots, birds, poultry, bird shops, or poultry markets. The clinical manifestations of psittacosis chlamydial pneumonia are not all specific. Most cases present with high fever, chills, headache, myalgia, cough, and pulmonary infiltration, as summarized in the above literature review. In severe cases, patients may progress to severe pneumonia and even have a poor prognosis (8), with some cases developing acute respiratory distress syndrome (ARDS). Furthermore, *Chlamydia psittaci* infection can impact multiple organ systems, leading to endocarditis, myocarditis, hepatitis, arthritis, keratoconjunctivitis, encephalitis, and ocular adnexal lymphoma (6, 8). Some patients may develop rhabdomyolysis, lower extremity venous thrombosis, lower extremity arteriosclerosis, pulmonary embolism, splenic infarction, and visual impairment. The present case presented with the formation of lower extremity venous thrombosis. Some cases may be accompanied by elevated blood glucose levels, with two documented instances in the literature review. Most patients exhibit normal white blood cell counts, elevated neutrophils, increased CRP levels, and elevated PCT levels. If systemic diseases are present, patients may present with abnormal liver function, hyponatremia, as well as elevated blood urea nitrogen and serum creatinine levels. Additionally, elevated D-dimer levels may also occur. The patient in this case presented with fever, cough, respiratory failure, leukocytosis, neutrophilia, and elevated levels of CRP, PCT, and D-dimer. The imaging manifestations of psittacosis chlamydial pneumonia are also not always specific. The majority of patients show high-density shadows and consolidation on chest imaging, while hilar lymph node enlargement and pleural effusion are relatively rare (8). High-resolution CT scans in some patients revealed that certain lesion areas presented with solid nodular and ground-glass opacities, bronchial signs, vacuolar signs, as well as halo reversal signs and migratory infiltrates. In this case, the patient displayed high-density consolidation shadows along with a small volume of right pleural effusion.

Severe pneumonia can be diagnosed through clinical manifestations and chest imaging; however, a definitive diagnosis of *Chlamydia psittaci* infection necessitates laboratory tests, including cell culture, serology, PCR, and NGS (8). Cell culture is conducted exclusively in biosafety level 3 (BSL-3) laboratories due to its high infectious potential. Serological tests exhibit cross-reactivity with other Chlamydia species, lack traceability, and demonstrate limited value for early diagnosis (6–8).

PCR (Polymerase Chain Reaction) stands for polymerase chain reaction. This technique features simple operation, rapid detection with results generally available within 1 to 2 h, alongside high sensitivity, strong specificity and low costs. At present, it is widely used in scenarios such as nucleic acid detection of infectious disease pathogens and screening of known gene mutations. Real-time polymerase chain reaction (RT-PCR) is an identification method with faster speed, higher sensitivity and better specificity (8). Nevertheless, polymerase chain reaction testing for *Chlamydia psittaci* is unavailable in most hospitals across China, including numerous Grade A tertiary hospitals. Such testing is only performed when clinicians highly suspect *Chlamydia psittaci* infection (8). The patient in this case was diagnosed via RT-PCR testing conducted by the local municipal Center for Disease Control and Prevention.

NGS refers to next-generation sequencing, also known as high-throughput sequencing. It can directly obtain pathogen information from lesioned tissues and body fluids, greatly shortening the detection cycle (8–10). NGS is capable of identifying unknown variants, novel pathogens, complex gene mutations and fusion genes, boasting irreplaceable advantages in precision medicine and the diagnosis of difficult and complicated cases. NGS also has several limitations, including the absence of universally recognized interpretation criteria, unclear correlation between sequencing results and treatment, potential contamination risks and relatively high costs. Moreover, its results need to be verified in combination with PCR or RT-PCR (8, 11).

In summary, these two molecular biology techniques each possess unique advantages and limitations in the diagnosis of severe pneumonia and can complement one another. Clinicians should rationally select the most appropriate diagnostic method based on the patient’s specific conditions, clinical indications, and the accessibility of different testing approaches.

This patient developed lower extremity deep vein thrombosis during a *Chlamydia psittaci* infection. However, when the patient does not exhibit typical symptoms such as hemoptysis, chest pain or leg swelling, early identification of venous thromboembolism (VTE) is particularly important. In this case, the patient’s D-dimer level was elevated upon admission, so an immediate lower extremity venous ultrasound examination was conducted, confirming deep vein thrombosis (DVT). Subsequently, the patient’s family agreed to undergo pulmonary artery CT (CTPA) examination, which indicated no concurrent pulmonary embolism.

During the management of this case, the patient initiated anticoagulation therapy immediately after diagnosis of deep vein thrombosis (DVT), followed by vena cava filter implantation and aggressive infection control with Omadacycline. This combination therapy strategy controlled the infection, prevented further thrombus expansion, and avoided related complications such as pulmonary embolism. While anticoagulation is essential for DVT management, it necessitates a careful assessment of risks and benefits (12). In this case, the patient initially received low molecular weight heparin (LMWH). After implantation of an intravenous filter, LMWH was used during the early phase, followed by a transition to oral rivaroxaban in the later stage. Compared with traditional anticoagulants, novel anticoagulants such as rivaroxaban, dabigatran, argatroban, bivalirudin, apixaban, and edoxaban exhibit stronger anticoagulant efficacy and lower bleeding risks, and have become the first-line drugs for antithrombotic therapy in recent years (12, 13). Rivaroxaban, in particular, offers greater convenience, a faster onset of action, and does not require monitoring, in contrast to warfarin (12, 14). Rivaroxaban can reduce thrombus volume and lower the risk of recurrence without increasing bleeding risk, making it more preferred than standard anticoagulants (12, 15).

The core pathological mechanism of venous thrombosis secondary to severe psittacosis is infection-induced systemic thromboinflammation: chlamydial virulence factors trigger massive release of pro-inflammatory factors, which damage vascular endothelium and activate coagulation pathways. Combined with risk factors such as hypoxia and bed rest, a hypercoagulable state is formed. This pathological process can be compared with SARS-CoV-2 and avian influenza infections: all three induce hypercoagulability via inflammatory storms, and venous thrombosis should be closely monitored in critically ill patients. However, SARS-CoV-2 can directly injure vascular endothelium, leading to a high incidence of arterial, venous and microvascular thrombosis; avian influenza mainly causes venous thrombosis through early severe hypoxia; psittacosis only stimulates endothelium indirectly via inflammation, with thrombosis limited to severe cases, predominantly deep venous thrombosis and pulmonary embolism, without high incidence of arterial or microvascular thrombosis, and coagulation disorders can be rapidly corrected after inflammation subsides. Drawing on the thrombosis prevention and treatment strategies for the two types of viral infections, early thrombosis screening and anticoagulant intervention for severe psittacosis are of great clinical significance.

Tetracycline derivatives, including traditional agents (doxycycline, minocycline), tigecycline and omadacycline, are first-line therapies for *Chlamydia psittaci* infection (6, 8). Omadacycline, a novel aminomethylcycline subclass of tetracyclines, represents a new oral/intravenous alternative distinct from early tetracyclines, doxycycline and minocycline. Tigecycline is another novel tetracycline-class glycylcycline antibiotic; unlike tigecycline (which is administered only intravenously and carries higher risks of gastrointestinal adverse reactions), omadacycline has dual intravenous and oral formulations and demonstrates superior clinical tolerability. Supplementary Table 1 shows that 44 patients received tetracycline therapy, among whom 41 achieved cure and one case showed improvement in visual symptoms after medication. However, the patient still died due to the underlying disease, indicating the definitive efficacy of tetracycline. Generally, a response such as a decrease in body temperature occurs within 24–48 h after treatment with tetracycline antibiotics (4). The treatment regimen should extend for at least 14 days, preferably 21 days, to mitigate the risk of recurrence (4, 6). For patients intolerant to tetracyclines, macrolides, such as azithromycin, represent the best alternative (4, 16). Fluoroquinolones, including moxifloxacin and levofloxacin, are also effective options (4). In cases of life-threatening infections, combination therapy involving tetracyclines, macrolides, and quinolones may be necessary (4). The patient in this case achieved favorable outcomes through targeted therapy involving intravenous tetracycline combined with fluoroquinolones. The successful treatment of this patient was attributed, on one hand, to the timely, precise, and potent administration of targeted medications, and on the other hand, to the management of comorbidities, including active anticoagulation therapy, prompt placement of a vena cava filter, and nutritional support.

In summary, *Chlamydia psittaci* infection poses diagnostic challenges due to low public awareness and diverse clinical manifestations. Definitive diagnosis relies on laboratory tests; PCR (particularly RT-PCR) is rapid and sensitive but remains underutilized, while NGS demonstrates advantages in complex cases, with the two methods complementing each other. The first-line treatment involves tetracyclines (e.g., omicacin), while severe cases may require combination therapy with macrolides and fluoroquinolones. Complications such as deep vein thrombosis necessitate prompt anticoagulation (with novel oral anticoagulants like rivaroxaban being superior) and caval filter placement. Enhanced clinical awareness of psittacosis, combined with precise diagnosis, standardized.

## Data Availability

The original contributions presented in the study are included in the article/Supplementary material, further inquiries can be directed to the corresponding author.
