# Supplementary material for: Severe Chlamydia psittaci pneumonia complicated by deep vein thrombosis: a case report and literature review
Source: Front Med (Lausanne). 2026 Jul 28;13:1865706. doi: 10.3389/fmed.2026.1865706 (PMC13457644; doi:10.3389/fmed.2026.1865706)
Supplement: Supplementary file 1 [file Data_Sheet_1.pdf]

Supplementary Table 1 Detailed information on the 54 case reports screened

Case	Gender	Age	Contact history	Clinical manifestations	Inflammatory markers	Imaging	Diagnostic method	Pre-diagnosis medications	Post-diagnosis medications	Outcome
1(4)	F	54	No bird contact	Headache, fever, dyspnea, fatigue, dry cough, nausea, memory loss, bilateral hearing loss, drowsiness	Normal WBC, elevated NEUT%, elevated CRP, elevated PCT	Chest CT revealed multiple patchy shadows in both lungs and bilateral pleural effusion.	NGS	Moxifloxacin	Doxycycline, moxifloxacin, azithromycin	Improved
2(17)	F	76	Poultry	Fever, dry cough	Elevated WBC, elevated NEUT%, elevated CRP, elevated PCT,	Chest CT revealed extensive areas of increased density in the right lung, accompanied by partial consolidation.	NGS	tigecycline	Moxifloxacin, tigecycline	Improved

3(18)	M	65	Unknown	Sudden hearing loss, cough with sputum, fever	Normal WBC, elevated NEUT%, elevated CRP	Chest CT revealed consolidation with inflammatory exudate in the left lung	NGS	Moxifloxacin	Tetracycline ,moxifloxacin	Improved
4(18)	M	61	Unknown	Sudden bilateral hearing loss, tinnitus, fever, headache, diarrhea	Unknown	Chest X-ray examination revealed consolidation in the right lung.	Serological test for Chlamydia psittaci is positive	Prednisolone	Erythromycin, amoxicillin	Improved
5(18)	F	15	Unknown	Recurrent interstitial keratitis and uveitis, sensorineural hearing loss, tinnitus, and vertigo	Unknown	Unknown	Chlamydia psittaci was cultured and isolated from conjunctival swabs.	Hormone eye drops	Doxycycline	Died
6(19)	M	26	Pigeon/chicken	Fever, cough	Normal WBC,	Chest CT revealed inflammat	NGS	levofloxacin	Omadacycline	Improved

			feces	with sputum	elevated NEUT%, elevated CRP, elevated PCT	ory infiltration in the right lower lobe of the lung with halo sign				
7(20)	M	16	Unknown	Cough, accompanied by scant sticky sputum, nasal congestion, and rhinorrhea	Normal	Chest CT revealed nodules in both lower lobes of the lungs and a small cavitation lesion in the right lower lobe.	NGS	Doxycycline	Doxycycline	Improved
8(21)	F	81	Unknown	Fever, cough with white sputum and copious volume、myalgia	Normal	Chest CT revealed inflammatory infiltration in the left lung with left alveolar consolidation	NGS	Moxifloxacin	Doxycycline	Improved
9(21)	M	45	Pigeon	Fever, expector	Normal WBC,	Chest CT revealed	NGS	levofloxaci	Doxycycline,	Impr

				ation, myalgia, fatigue	elevated NEUT%, elevated CRP, elevated PCT	right lung consolidation and diffuse infiltration in both lungs		n	moxifloxacin	oved
10(21)	F	85	Bird-exposed	Fever, expectoration, headache, myalgia, vomiting	Normal WBC, elevated NEUT%, elevated CRP, elevated PCT	Chest CT shows consolidation in the right lung	NGS	Ertapenem, biapenem, teicoplanin	Doxycycline, moxifloxacin	Improved
11(21)	F	66	Poultry	Fever, chills, dry cough, dizziness	Normal WBC, normal NEUT%, elevated CRP, normal PCT	Chest CT revealed a solid mass in the right lung	NGS	Levofloxacin	Moxifloxacin	Improved
12(21)	F	61	parrot	Fever, chills, fatigue, expectoration, and dizzines	Normal WBC, normal NEUT%, elevated CRP,	Chest CT revealed patchy infiltration and consolidation in both lungs	NGS	Moxifloxacin	Moxifloxacin	Improved

				s	normal PCT					
13(22)	M	54	Unknown	Cough, sputum, fever, dyspnea	Elevated WBC, NEUT% unknown,elevated CRP, elevated PCT	Chest CT revealed scattered patchy high-density shadows and consolidation in the upper lobes of both lungs, predominantly in the right lung.	NGS	tigecycline	Tigecycline, Azithromycin	Improved
14(23)	M	64	Chicken	Fever, cough, chest tightness	Normal WBC,other unknown	Chest CT revealed patchy hyperechoic shadows with blurred margins in the left lung. Patchy exudative lesions were observed in the left lung, accompanied by a	NGS	moxifloxacin	Doxycycline	Improved

						small amount of left-sided pleural effusion and migratory infiltrates.				
15(24)	M	62	Parrot	febrile, headache, myalgia, cough with yellow sputum	Elevated WBC,other unknown	The chest X-ray revealed consolidation in the lower left lung.	PCR	doxycycline	doxycycline	Improved
16(24)	M	55	chicken	Fever, headache, bone pain, cough	Normal WBC,other unknown	The chest X-ray revealed a shadow in the right upper lung field.	PCR	doxycycline	doxycycline	Improved
17(24)	F	42	goose/chicken	Fever, cough, expectoration of yellow sputum with blood, and dyspnea	Normal WBC,other unknown	Chest X-ray revealed consolidation in the middle and lower right lung, with	PCR	doxycycline	doxycycline	Improved

						patchy opacities in the left lung.				
18(25)	M	48	Pigeon	Cough with scant yellow sputum, dyspnea, fever, chills, and sweating	Elevated WBC, elevated NEUT%, elevated CRP, elevated PCT	The chest CT shows diffuse, bilateral, and mass-like infiltration.	NGS	Moxifloxacin	Doxycycline, moxifloxacin	Improved
19(26)	F	47	Chicken/duck	Fever, cough, dyspnea, headache, chills, fatigue、anorexia	Normal WBC, elevated NEUT%, elevated CRP, elevated PCT	Chest CT revealed multiple patchy exudative opacities in both lungs, accompanied by extensive consolidation in the right lower lung.	NGS	levofloxacin	Levofloxacin ,doxycycline	Improved
20(27)	M	41	pigeon	Fever, sore throat, cough, yellow	Normal WBC, elevated NEUT	Chest CT revealed bilateral pneumonia	NGS	moxifloxacin	moxifloxacin	Improved

				sputum	%, elevated CRP, normal PCT					
21(28)	Unknown	Unknown	Parrot	Chills, fever, dry cough, headache, diarrhea, vomiting	Unknown	Chest X-ray revealed pneumonia in the right lung.	SeroFIAT Chlamydia Immunofluorescence Detection (MIF) Kit	azithromycin	azithromycin	Improved
22(29)	M	62	Undercooked pigeon	Fever, severe diarrhea, dry cough	Elevated WBC, elevated NEUT%, CRP unknown, elevated PCT	Chest CT revealed severe inflammation and consolidation in the right lower lung.	PCR	Cephalosporin	Levofloxacin, doxycycline	Improved
23(30)	M	83	Pigeon	Fever, chills, cough, scanty sputum,	Elevated WBC, elevated	Chest CT revealed right lung consolidation and bilateral pleural	NGS	vancomycin	Levofloxacin , minocycline	Improved

				chest tightness, drowsiness	NEUT %, elevated CRP, normal PCT	effusion, with more pronounced right-sided involvement.				
24(30)	M	70	Parrot	Fever, chills, anorexia, dry cough	Normal WBC, elevated NEUT %, elevated CRP, normal PCT	Chest CT revealed left lung exudative shadow, minimal pleural effusion, scattered calcification foci in both lungs, and slight interstitial changes in the right lung.	NGS	None	Moxifloxacin	Improved
25(30)	F	66	Parrot	Fever, cough, sore throat, fatigue	Normal WBC, elevated NEUT %, elevated CRP,	Chest CT revealed multiple patchy ground-glass opacities in both	NGS	Piperacillin-sulbactam	Levofloxacin , minocycline	Improved

					normal PCT	lungs, with patchy consolidation in the right lower quadrant. Bronchial airway signs were observed within the consolidation, along with minimal pleural effusion and pericardial effusion.				
26(31)	M	44	Parrot	Intermittent fever, severe dry cough, headache, epigastric pain, dyspnea	Elevated WBC,other unknown	Chest CT revealed infiltrates in the upper left lung and patchy reticular infiltrates in the left lung.	Chlamydia IgM/IgG/IgA MIF Kit	minocycline	Minocycline	Improved
27(12)	M	51	Poultry	Fever, cough,	Elevated	Chest CT revealed	NGS	Cefoperazone-sulbac	doxycyclin	Impr

				dyspnea, shortness of breath	WBC, elevated NEUT%, elevated CRP, elevated PCT	extensive consolidation in both lungs with minimal right-sided pleural effusion.		tam	e	oved
28(32)	M	56	Feathers and feces of infected birds	Fever, dizziness, fatigue, cough, white sputum, chest tightness	Normal WBC, elevated NEUT%, elevated CRP,PCT unknown	Chest CT revealed consolidation in the right lung with bronchial air-fluid filling and air-fluid space signs within the lesion, minimal exudate and ground-glass opacities in the left lung, and trace pleural effusion.	NGS	Levofloxacin	moxifloxacin	Improved
29(33)	M	64	Duck	Nausea, fatigue, myalgia, chills, high	Elevated WBC,	Chest CT revealed multiple patchy opacities	NGS	moxifloxacin	Moxifloxacin	Improved

				fever, dyspnea	elevated NEUT%, elevated CRP, elevated PCT	with blurred margins in both lungs; extensive patchy areas of increased density in the left lung with partial lobar consolidation; significant consolidation in the left lung and exudative lesions in the right lung; infectious lesions in both lungs, with marked involvement in the left lung.				
30(34)	F	71	pigeons	Fever, dyspnea, chills, fatigue, dry	Elevated WBC, elevated	Chest CT revealed left lung pneumonia and mediastinal	NGS	levofloxacin	Tigecycline, levofloxacin	Improved

				cough, nausea, vomiting, diarrhea	NEUT%, elevated CRP, elevated PCT	lymphadenopathy				
31(35)	F	67	Poultry	Fever, dyspnea, diarrhea	Normal WBC, elevated NEUT%, elevated CRP, elevated PCT	Chest CT revealed multiple patchy lesions in both lungs, with more pronounced manifestations in the left lung, accompanied by left pleural effusion.	NGS	levofloxacin	doxycycline	Improved
32(35)	M	64	Frequenting the market	Fever, cough, dizziness	Normal WBC, elevated NEUT%, elevated CRP, elevate	Chest CT revealed multiple patchy shadows in both lungs, consolidation in the left lung, and pleural	NGS	moxifloxacin	doxycycline	Improved

					d PCT	effusion in both lungs.				
33(35)	M	48	Poultry	Fever, cough, sputum	Normal WBC, normal NEUT %, elevated CRP, normal PCT	Chest CT revealed multiple patchy ground-glass opacities and reticular opacities in the right lung.	NGS	Meropenem	doxycycline	Improved
34(35)	F	66	Poultry	Fever, cough, fatigue	Normal WBC, elevated NEUT %, elevated CRP, normal PCT	Chest CT revealed patchy high-density shadows in both lungs and pleural effusion in the left lung.	NGS	tigecycline	tigecycline	Improved
35(35)	F	66	swallow	Fever, cough, diarrhea	Normal WBC, elevated NEUT %,	Chest CT revealed multiple patchy shadows and consolidat	NGS	moxifloxacin	doxycycline	Improved

					elevated CRP, elevated PCT	ion in both lungs, with pleural effusion in both lungs.				
36(35)	M	43	pigeon	Fever, cough, diarrhea	Elevated WBC, elevated NEUT%, elevated CRP, elevated PCT	Chest CT revealed scattered patchy and nodular high-density opacities in both lungs, with pleural effusion in both lungs.	NGS	moxifloxacin	Levofloxacin , doxycycline	Improved
37(35)	F	72	chicken	Fever, cough, sputum	Normal WBC, normal NEUT%, elevated CRP, elevated PCT	Chest CT revealed multiple nodules in both lungs and pleural effusion in both lungs	NGS	,tigecycline	doxycycline	Improved
38(35)	F	50	Poultry	Fever,	Elevat	Chest CT revealed	NGS	moxifloxaci	doxycyclin	Impr

				dyspnea, vomiting	ed WBC, elevated NEUT%, elevated CRP, elevated PCT	patchy consolidation in the right lung with air bronchogram sign and pleural effusion in both lungs.		n	e	oved
39(35)	M	54	Poultry	Fever, cough, diarrhea	Normal WBC, elevated NEUT%, elevated CRP, elevated PCT	Chest CT revealed multiple patchy shadows in both lungs and pleural effusion in the left lung.	NGS	levofloxacin	doxycycline	Died
40(35)	F	69	Parrot	Fever, diarrhea	Normal WBC, elevated NEUT%, elevated CRP, elevated PCT	Chest CT revealed multiple patchy and ground-glass opacities in both lungs, with pleural	NGS	moxifloxacin	Not applicable	Died

						effusion in both lungs.				
41(36)	F	33	Pigeon	Fever, chills, cough, chest tightness, lower abdominal pain	Normal WBC, normal NEUT %, elevated CRP, elevated PCT	Chest CT revealed ground-glass opacities in both lungs, air bronchogram sign, and a small amount of pleural effusion in the right lung.	NGS	moxifloxacin	Azithromycin	Improved
42(36)	M	59	Chicken	Fever, chills, myalgia, anorexia	Normal WBC, normal NEUT %, elevated CRP, elevated PCT	Chest CT revealed ground-glass opacities in both lungs, air bronchogram sign, and a small amount of pleural effusion in the right lung.	NGS	moxifloxacin , vancomycin	Vancomycin, azithromycin	Improved

43(36)	M	61	Parrot	Fever, cough, sputum, sore throat, anorexia	Normal WBC, normal NEUT %, elevated CRP, elevated PCT	Chest CT revealed right lung consolidation with ground-glass opacities, air bronchogram sign, and minimal pleural effusion in the right lung.	NGS	Moxifloxacin	Minocycline	Improved
44(36)	F	61	Parrot	Fever, chills, dry cough, sputum, chest tightness, fatigue, headache, chest pain, myalgia, anorexia	Elevated WBC, elevated NEUT %, elevated CRP, elevated PCT	Chest CT revealed right lung consolidation with ground-glass opacities, air bronchogram sign, and minimal pleural effusion in the right lung.	NGS	moxifloxacin	Moxifloxacin, minocycline	Improved
45(36)	F	31	None	Fever, chills, cough,	Normal WBC, elevate	Chest CT revealed right lung consolidat	NGS	moxifloxacin	Moxifloxacin, minocyclin	Improved

				chest tightness, fatigue, headache, sore throat, myalgia, anorexia	d NEUT %, elevated CRP, elevated PCT	ion with ground-glass opacities, air bronchogram sign, and minimal pleural effusion in both lungs.			e	
46(36)	M	56	Duck	Fever , chills, cough, chest tightness, fatigue, myalgia, anorexia	Normal WBC, elevated NEUT %, elevated CRP, elevated PCT	Chest CT revealed right lung consolidation with ground-glass opacities, air bronchogram sign, and small amount of pleural effusion in the left lung.	NGS	moxifloxacin	Moxifloxacin, minocycline	Improved
47(36)	F	59	Parrot	Fever chills, cough, sputum, fatigue, headach	Normal WBC, elevated NEUT %,	Chest CT revealed right lung consolidation with ground-glass opacities,	NGS	Moxifloxacin	Moxifloxacin, minocycline	Improved

				e, myalgia, anorexia	elevated CRP, elevated PCT	air bronchogram sign, minimal pleural effusion in the left lung, and enlarged lymph nodes and spleen.				
48(36)	M	67	Pigeon	Fever , cough, scanty sputum	Normal WBC, normal NEUT %, elevated CRP, elevated PCT	Chest CT revealed ground-glass opacities with consolidation in both lungs, air bronchogram sign, minimal pleural effusion in the left lung, and lymphadenopathy.	NGS	Piperacillin-tazobactam	Levofloxacin , minocycline	Improved
49(37)	M	68	None	Fever; headache; chills; cough; bloody sputum	Normal WBC, elevated NEUT %,	Chest CT revealed patchy high-density shadows	NGS	Cephalosporin, piperacillin-tazobactam, oseltamivir,	Doxycycline,	Improved

					elevated CRP, elevated PCT	in the right upper lung and a small amount of pleural effusion in the right thoracic cavity.		meropenem		
50(37)	F	69	Chicken	Fever, chest tightness, tachypnea, night sweats, dry cough	Normal WBC, elevated NEUT%, elevated CRP, elevated PCT	Chest CT revealed patchy and mass-like high-density shadows in the left lung, accompanied by air bronchogram signs.	NGS	levofloxacin	Doxycycline	Improved
51(37)	M	51	None	Chest pain; fever; cough with sputum production; blood-tinged sputum;	Elevated WBC, elevated NEUT%, elevated CRP, normal	Chest CT revealed multiple patchy and mass-like high-density opacities in both lungs,	NGS	levofloxacin	Doxycycline	Improved

				joint swelling and joint pain	PCT	accompanied by localized consolidation and air bronchogram signs, with minimal bilateral pleural effusion.				
52(37)	M	69	Chicken/duck	Fever, chills, dry cough, fatigue, night sweats	Normal WBC, elevated NEUT%, elevated CRP, elevated PCT	Chest CT revealed patchy high-density shadows in the right lung, accompanied by localized consolidation and air bronchogram signs.	NGS	levofloxacin	Doxycycline	Improved
53(37)	M	63	Chicken	Cough, sputum, fever, chills, fatigue, anorexia	Normal WBC, normal NEUT%, elevated CRP,	Chest CT revealed large patchy areas of high-density	NGS	moxifloxacin	Doxycycline	Improved

					elevated PCT	shadows in the upper left lung with localized consolidation; minimal consolidation shadows in the lower left lung, and a small amount of pleural effusion in the left thoracic cavity.				
54(37)	F	58	Chicken/duck	Fever, chills, dizziness, dry cough	Normal WBC, elevated NEUT%, elevated CRP, elevated PCT	Chest CT revealed a mass-like high-density shadow in the upper left lung, accompanied by consolidation and air bronchogram sign.	NGS	levofloxacin	Doxycycline	Improved

NEUT%,Neutrophil Percentage

References for Supplementary Table 1

4.Shi Y, Chen J, Shi X, Hu J, Li H, Li X, et al. A case of chlamydia psittaci caused severe pneumonia and meningitis diagnosed by metagenome next-generation sequencing: a case report and literature review. BMC Infect Dis. 2021 Jun 30;21(1):621. doi:10.1186/s12879-021-06205-5.

12.Zhang A, Huang T, Lao X, Ma J, Xia X, Liang J. Severe Chlamydia psittaci pneumonia complicated with deep vein thrombosis: a case report. Front Med (Lausanne). 2025 Feb 25;12:1527556. doi:10.3389/fmed.2025.1527556.

17.Yu Z, Qian Y, Lv L, Hu W. A case of severe psittacosis pneumonia complicated by splenic infarction. BMC Infect Dis. 2024 Oct 25;24(1):1203. doi:10.1186/s12879-024-10111-x.

18.Wu HH, Zhao PP, Wang XM, Ren YY, Fang SY. Hearing loss as the first symptom of pneumonia caused by Chlamydia psittaci: a case report and literature review. BMC Infect Dis. 2024 Nov 18;24(1):1314. doi:10.1186/s12879-024-10210-9.

19.Zhang S, Fu Y, Fang L, Xu Q, Gu S, Zhou H, et al. Psittacosis pneumonia with the reversed halo sign: a case report and literature review. BMC Infect Dis. 2025 May 17;25(1):717. doi:10.1186/s12879-025-11081-4.

20.Zhong H, Huang D, Lin Y, Yang P, Shi H, Yang S, et al. Chlamydia psittaci pneumonia manifested as pulmonary nodules and cavitary lesions: a case report. BMC Infect Dis. 2025 Sep 29;25(1):1215. doi:10.1186/s12879-025-11676-x.

21.Gu L, Liu W, Ru M, Lin J, Yu G, Ye J, et al. The application of metagenomic next-generation sequencing in diagnosing Chlamydia psittaci pneumonia: a report of five cases. BMC Pulm Med. 2020 Mar 17;20(1):65. doi:10.1186/s12890-020-1098-x.

22.Shao S, Liu J, Wu Z, Wu S. Chlamydophila psittaci pneumonia followed by lower gastrointestinal ischemic necrosis: a case report. Front Med (Lausanne). 2025 Jan 8;11:1394897. doi:10.3389/fmed.2024.1394897.

23.Wang J, Zhu Y, Mo Q, Yang Y. Case Report: A Chlamydia psittaci pulmonary infection presenting with migratory infiltrates. Front Public Health. 2022 Dec 19;10:1028989. doi:10.3389/fpubh.2022.1028989.

24.Chau S, Tso EY, Leung WS, Fung KS. Three cases of atypical pneumonia caused by Chlamydophila psittaci. Hong Kong Med J. 2015 Jun;21(3):272–275. doi:10.12809/hkmj144321.

25.He Y, Wang S, Deng J, Pu Q, Chen H, Huang L. Chlamydia psittaci pneumonia complicated with lower extremity atherosclerotic occlusive disease. Infect Drug Resist. 2023 Apr 12;16:2141–2145. doi:10.2147/IDR.S393256.

26.Fang C, Xu L. Chlamydia psittaci pneumonia-induced pulmonary thrombosis: a case report. Infect Drug Resist. 2023 Nov 6;16:7063–7069. doi:10.2147/IDR.S435246.

27.Zhang A, Lao X, Liang J, Xia X, Ma L, Liang J. Case Report: Pneumonia caused by Chlamydia psittaci and Cryptococcus co-infection. Infect Drug Resist. 2024 Mar 6;17:845–849. doi:10.2147/IDR.S445920.

28.Chaber AL, Jelocnik M, Woolford L. Undiagnosed cases of human pneumonia following exposure to Chlamydia psittaci from an infected rosella parrot. Pathogens. 2021 Jul 30;10(8):968. doi:10.3390/pathogens10080968.

29.Deng H, Liang X, Dang S, Qiu J. Chlamydia psittaci enteric infection in an adult man: a case report. Int J Infect Dis. 2025 Sep;158:107967. doi:10.1016/j.ijid.2025.107967.

30.Xu J, Sheng HY, Lu XX, Liu XF. [Psittacosis pneumonia with pleural effusion: report of three cases and literature review]. Zhongguo Yi Xue Ke Xue Yuan Xue Bao. 2022 Oct;44(5):923–928. doi:10.3881/j.issn.1000-503X.14760.

31.Cheng YJ, Lin KY, Chen CC, Huang YL, Liu CE, Li SY. Zoonotic atypical pneumonia due to Chlamydophila psittaci: first reported psittacosis case in Taiwan. J Formos Med Assoc. 2013 Jul;112(7):430–433. doi:10.1016/j.jfma.2012.08.017.

32.Liu GF, Cui P, Huang JJ. [One case report of Chlamydia psittaci pneumonia]. Zhonghua Lao Dong Wei Sheng Zhi Ye Bing Za Zhi. 2022 Apr 20;40(4):300–303. doi:10.3760/cma.j.cn121094-20210330-00176.

33.Qi YF, Huang JL, Chen JH, Huang CP, Li YH, Guan WJ. [Chlamydia psittaci pneumonia complicated with rhabdomyolysis: a case report and literature review]. Zhonghua Jie He He Hu Xi Za Zhi. 2021 Sep 12;44(9):806–811. doi:10.3760/cma.j.cn112147-20210228-00137.

34.Liu J, Gao Y. Tigecycline in the treatment of severe pneumonia caused by Chlamydia psittaci: a case report and literature review. Front Med (Lausanne). 2022 Nov 24;9:1040441. doi:10.3389/fmed.2022.1040441.

35.Zheng X, Wu C, Jiang B, Qin G, Zeng M. Clinical analysis of severe Chlamydia psittaci pneumonia: case series study. Open Life Sci. 2023 Sep 26;18(1):20220698. doi:10.1515/biol-2022-0698.

36.Zhang Y, Jiang X, Ye W, Sun J. Clinical features and outcome of eight patients with Chlamydia psittaci pneumonia diagnosed by targeted next generation sequencing. Clin Respir J. 2023 Sep;17(9):915–930. doi:10.1111/crj.13681.

37.Dai J, Lian X, Mo J, Li X, Mo W, Wang H, et al. Case report: a clinical case study of six patients with Chlamydia psittaci pneumonia. Front Cell Infect Microbiol. 2023 Feb 24;13:1084882. doi:10.3389/fcimb.2023.1084882.
